# Virtual screening of GPCRs: An *in silico* chemogenomics approach

**DOI:** 10.1186/1471-2105-9-363

**Published:** 2008-09-06

**Authors:** Laurent Jacob, Brice Hoffmann, Véronique Stoven, Jean-Philippe Vert

**Affiliations:** 1 Mines ParisTech, Centre for Computational Biology, 35 rue Saint-Honoré, F-77305, Fontainebleau, France; 2 Institut Curie, Paris, F-75248, France; 3 INSERM, U900, Paris, F-75248, France

## Abstract

**Background:**

The G-protein coupled receptor (GPCR) superfamily is currently the largest class of therapeutic targets. *In silico *prediction of interactions between GPCRs and small molecules in the transmembrane ligand-binding site is therefore a crucial step in the drug discovery process, which remains a daunting task due to the difficulty to characterize the 3D structure of most GPCRs, and to the limited amount of known ligands for some members of the superfamily. Chemogenomics, which attempts to characterize interactions between all members of a target class and all small molecules simultaneously, has recently been proposed as an interesting alternative to traditional docking or ligand-based virtual screening strategies.

**Results:**

We show that interaction prediction in the chemogenomics framework outperforms state-of-the-art individual ligand-based methods in accuracy both for receptor with known ligands and without known ligands. This is done with no knowledge of the receptor 3D structure. In particular we are able to predict ligands of orphan GPCRs with an estimated accuracy of 78.1%.

**Conclusion:**

We propose new methods for *in silico *chemogenomics and validate them on the virtual screening of GPCRs. The methods represent an extension of a recently proposed machine learning strategy, based on support vector machines (SVM), which provides a flexible framework to incorporate various information sources on the biological space of targets and on the chemical space of small molecules. We investigate the use of 2D and 3D descriptors for small molecules, and test a variety of descriptors for GPCRs. We show that incorporating information about the known hierarchical classification of the target family and about key residues in their inferred binding pockets significantly improves the prediction accuracy of our model.

## Background

The G-protein coupled receptor (GPCR) superfamily is comprised of an estimated 600–1,000 members and is the largest known class of molecular targets with proven therapeutic value. They are ubiquitous in our body, being involved in regulation of every major mammalian physiological system [1], and play a role in a wide range of disorders including allergies, cardiovascular dysfunction, depression, obesity, cancer, pain, diabetes, and a variety of central nervous system disorders [2-4]. They are integral membrane proteins sharing a common global topology that consists of seven transmembrane alpha helices, an intracellular C-terminal, an extracellular N-terminal, three intracellular loops and three extracellular loops. There are four main classes of GPCRs (A, B, C and D) defined in terms of sequence similarity [5]. Their location on the cell surface makes them readily accessible to drugs, and 30 GPCRs are the targets for the majority of best-selling drugs, representing about 40% of all prescription pharmaceuticals on the market [6]. Besides, the human genome contains several hundreds unique GPCRs which have yet to be assigned a clear cellular function, suggesting that they are likely to remain an important target class for new drugs in the future [7]. Predicting interactions *in silico *between small molecules and GPCRs is not only of particular interest for the drug industry, but also a useful step for the elucidation of many biological process. First, it may help to decipher the function of so-called *orphan *GPCRs, for which no natural ligand is known. Second, once a particular GPCR is selected as a target, it may help in the selection of promising molecule candidates to be screened *in vitro *against the target for lead identification.

*In silico *virtual screening of GPCRs is however a daunting task, both for receptor-based approaches (also called docking) and for ligand-based approaches. The former relies on the prior knowledge of the 3D structure of the protein, in a context where only two GPCR structures are currently known (bovine rhodopsin and human *β*_2_-adrenergic receptor). Indeed, GPCRs, like other membrane proteins, are notoriously difficult to crystallize. As a result, docking strategies for screening small molecules against GPCRs are often limited by the difficulty to model correctly the 3D structure of the target. To circumvent the lack of experimental structures, various studies have used 3D structural models of GPCRs built by homology modeling using bovine rhodopsin as a template structure. Docking a library of molecules into these modeled structures allowed the recovery of known ligands [8-11], and even identification of new ligands [12,13]. However, docking methods still suffer from docking and scoring inaccuracies, and homology models are not always reliable-enough to be employed in target-based virtual screening. Methods have been proposed to enhance the quality of the models for docking studies by global optimization and flexible docking [9], or by using different sets of receptor models [11]. Nevertheless, these methods have been applied only to class A receptors and they are expected to show limited performances for GPCRs sharing lower sequence similarity with rhodopsin, especially in the case of receptors belonging to classes B, C and D. Alternatively, ligand-based strategies, in particular quantitative structure-activity relationship (QSAR), attempt to predict new ligands from previously known ligands, often using statistical or machine learning approaches. Ligand-based approaches are interesting because they do not require the knowledge of the target 3D structure and can benefit from the discovery of new ligands. However, their accuracy is fundamentally limited by the amount of known ligands, and degrades when few ligands are known. Although these methods were successfully used to retrieve strong GPCR binders [14], they are efficient for lead optimization within a previously identified molecular scaffold, but are not appropriate to identify new families of ligands for a target. At the extreme, they cannot be pursued for the screening of orphan GPCRs. In this paper, we present a contribution to the screening of GPCRs, that is complementary to the above docking and ligand-based approaches. The method is related to ligand-based approaches, but because it allows to share information between different GPCRs, it can be used for orphan GPCRs, possibly in parallel to docking methods in order to increase the prediction quality.

Indeed, instead of focusing on each individual target independently from other proteins, a recent trend in the pharmaceutical industry, often referred to as *chemogenomics*, is to screen molecules against several targets of the same family simultaneously [15,16]. This systematic screening of interactions between the chemical space of small molecules and the biological space of protein targets can be thought of as an attempt to fill a large 2D *interaction matrix*, where rows correspond to targets, columns to small molecules, and the (*i*, *j*)-th entry of the matrix indicates whether the *j*-th molecule can bind the *i*-th target. While in general the matrix may contain some description of the strength of the interaction, such as the association constant of the complex, we will focus in this paper on a simplified description that only differentiates binding from non-binding molecules, which results in a binary matrix of target-molecule pairs. This matrix is already sparsely filled with our current knowledge of protein-ligand interactions, and chemogenomics attempts to fill the holes. While classical docking or ligand-based virtual screening strategies focus on each single row independently from the others in this matrix, *i.e.*, treat each target independently from each others, the chemogenomics approach is motivated by the observation that similar molecules can bind similar proteins, and that information about a known interaction between a ligand and a GPCR could therefore be a useful hint to predict interaction between similar molecules and similar GPCRs. This can be of particular interest when, for example, a particular target has few or no known ligands, but similar proteins have many: in that case it is tempting to use the information about the known ligands of similar proteins for a ligand-based virtual screening of the target of interest. In this context, we can formally define *in silico *chemogenomics as the problem of predicting interactions between a molecule and a ligand (*i.e.*, a hole in the matrix) from the knowledge of all other known interactions or non-interactions (*i.e.*, the known entries of the matrix).

Recent reviews [15-18] describe several strategies for *in silico *chemogenomics. A first class of approaches, called *ligand-based chemogenomics *by [18], pool together targets at the level of families (such as GPCR) or subfamilies (such as purinergic GPCR) and learn a model for ligands at the level of the family [19,20]. Such strategies could be facilitated by the design of libraries of annotated ligands [21]. Other approaches, termed *target-based chemogenomic *approaches by [18], cluster receptors based on ligand binding site similarity and again pool together known ligands for each cluster to infer shared ligands [22]. Finally, a third strategy termed *target-ligand *approach by [18] attempts to predict ligands for a given target by leveraging binding information for other targets in a single step, that is, without first attempting to define a particular set of similar receptors. This strategy was pioneered by [23]. [24] predicted ligands of orphan GPCR. They merged descriptors of ligands and targets to describe putative ligand-receptor complexes, and used SVM to discriminate real complexes from ligand-receptors pairs that do not form complexes. A similar approach termed *proteochemometrics *was used in [25,26] to correlate ligand-receptor descriptions to the corresponding binding affinities. [27] followed a similar idea to [24] with different descriptors, and showed in particular that the SVM formulation allows to generalize the use of vectors of descriptors to the use of positive definite kernels to describe the chemical and the biological space in a computationally efficient framework. [27] were not able to show, however, significant benefits with respect to the individual approach that learns a separate classifier for each GPCR (except in the case of orphan GPCRs, for which their approach performed better than the baseline random classifier). Recently, in the context of predicting interactions between peptides and different alleles of MHC-I molecules, [28] followed a similar approach and highlighted the importance of choosing adequate descriptors for small molecules and targets. They obtained state-of-the-art prediction accuracy for most MHC-I allele, in particular for those with few known binding peptides. [29] on the other hand applied this approach to predict interaction between various potential targets including GPCRs, enzymes and ion channels. Using general descriptors for targets, they obtained predictors that were more accurate than state-of-the-art individual methods both for the orphan targets and for the targets for which some ligands were already known.

In this paper we go one step further in this direction and present an *in silico *chemogenomics approach specifically tailored for the screening of GPCRs, although the method could in principle be adapted to other classes of therapeutic targets. We follow the idea of [24] and the algorithmic trick of [27], which allows us to systematically test a variety of descriptors for both the molecules and the GPCRs. We test 2D and 3D descriptors to describe molecules, and five ways to describe GPCRs, including a description of their relative positions in current hierarchical classifications of the superfamily, and information about key residues likely to be in contact with the ligand. We evaluate the performance of all combinations of these descriptions on the data of the GLIDA database [30], which contains 34686 reported interactions between human GPCRs and small molecules, and observe that the choice of the descriptors has a significant impact on the accuracy of the models. However, in all cases, we obtained significant improvements of the prediction accuracy with respect to the individual learning setting.

### Data

We used the GLIDA GPCR-ligand database [30] which includes 22964 known ligands for 3738 GPCRs from human, rat and mouse. The ligand database contains highly diverse molecules, from ions and very small molecules up to peptides, and a significant number of duplicates. These redundancies were eliminated. Elimination of duplicates present in the GLIDA database was important here because it could have led to over-optimistic evaluation in the cross-validation procedure described below. The remaining molecules were further filtered in order to satisfy two constraints. First, our method relies on the evaluation of similarities between molecules using kernels, which makes sense only if the molecules are comparable in size. Second, since the long term goal is to identify drug candidates targeting GPCRs, it was important to retain drug-like compounds, i.e. molecules having the adequate physico-chemical characteristics to be potential drugs candidates satisfying ADME criteria [31]. Therefore, to only keep drug-like compounds, we filtered the GLIDA database using the filter program (OpenEye Scientific Software) with standard parameters, which removes molecules according to calculated properties such as molecular weight, hydrogen bond donor and acceptor count, number of rotatable bonds, ring size and number etc... as discussed in [32-35]. For example, only molecules of molecular weights ranging from 150 Da to 450 Da were kept (the classically accepted range for drugs), since the aim was to evaluate if statistical learning was possible on drug-like compounds. Another example was the elimination of molecules with more than 10 rotatable bonds (although most of them being already filtered out on the molecular weight criterion). Indeed, they correspond to very flexible molecules that are not suitable for the use of 3D descriptors. Overall these filters retained 2446 molecules, available under a 2D description file in the GLIDA data bank, and giving 4051 interactions with the human GPCRs. The number of molecules retained is only a small fraction of the GLIDA database, but it corresponds to all drug-like compounds of this database. For each positive interaction given by this restricted set, we generated a negative interaction involving the same receptor and one of the ligands that was in the database and that was not indicated as one of its ligands. This may have generated a few false negative points in our benchmark, and it would be interesting to use experimentally tested negative interactions. However, the mean similarity between the different ligands in the database using the Tanimoto kernel, a classical normalized similarity measure for ligands which is later used in our method, is quite low (0.13). Besides, only 6.7% of the ligands have a mean similarity of more than 0.2 to the other ligands. This suggests that even if false negative have to be expected, this method to generate negative interaction is a reasonable approximation. We loaded the sequences of all GPCRs that are able to bind any of these ligands, which resulted in 80 sequences, all corresponding to human GPCRs. The retained GPCRs were significantly diverse in sequence, most of them sharing 15% to 50% pairwise sequence similarities. Furthermore, they belong to various families, according to the GLIDA classification. They are found in several subfamilies of class A (rhodopsin-like receptors), classes B (secretin family) and C (metabotropic family). In the GLIDA database, GPCRs are classified in hierarchy (as mentioned above) which was also loaded for use in the hierarchy kernel.

## Methods

In this section, we first review the methods proposed by [24,27] for *in silico *chemogenomics with SVM, before presenting the particular descriptors we propose to use for molecules and GPCRs within this framework.

### In silico chemogenomics with machine learning

We consider the problem of predicting interactions between GPCRs and small molecules. For this purpose we assume that a list of target/small molecule pairs {(*t*_1_, *m*_1_),...,(*t*_*n*_, *m*_*n*_)}, known to interact or not, is given. Such information is often available as a result of systematic screening campaigns in the pharmaceutical industry, or on dedicated databases. Our goal is then to create a model to predict, for any new candidate pair (*t*, *m*), whether the small molecule *m *is likely to bind the GPCR *t*.

A general method to create the predictive model is to follow these four steps:

1. Choose *n*_*tar *_descriptors to represent each GPCR target *t *in the biological space by a *n*_*tar*_-dimensional vector $\Phi_{tar}\left(t\right)=\left(\Phi_{tar}^{1}\left(t\right),...,\Phi_{tar}^{n_{tar}}\left(t\right)\right)$;

2. In parallel, choose *n*_*mol *_descriptors to represent each molecule *m *in the chemical space by a *n*_*mol*_-dimensional vector $\Phi_{mol}\left(m\right)=\left(\Phi_{mol}^{1}\left(m\right),...,\Phi_{mol}^{n_{mol}}\left(m\right)\right)$;

3. Derive a vector representation of a candidate target/molecule complex Φ_*pair*_(*t*, *m*) from the representations of the target Φ_*tar*_(*t*) and of the molecule Φ_*mol*_(*m*);

4. Use a statistical or machine learning method to train a classifier able to discriminate between binding and non-binding pairs, using the training set of binding and non-binding pairs {Φ_*pair*_(*t*_1_, *m*_1_),..., Φ_*pair*_(*t*_*n*_, *m*_*n*_)}

While the first two steps (selection of descriptors) may be specific to each particular chemogenomics problem, the last two steps define the particular strategy used for *in silico *chemogenomics. For example, [24,36] proposed to concatenate the vectors Φ_*tar*_(*t*) and Φ_*mol*_(*m*) to obtain a (*n*_*tar *_+ *n*_*mol*_)-dimensional vector representation of the ligand-target complex Φ_*pair*_(*t*, *m*), and to use a SVM as a machine learning engine. [27] followed a slightly different strategy for the third step, by forming descriptors for the pair (*t*, *m*) as *product *of small molecule and target descriptors. More precisely, given a molecule *m *described by a vector Φ_*mol*_(*m*) and a GPCR *t *described by a vector Φ_*tar*_(*t*), the pair (*t*, *m*) is represented by the tensor product:

(1) Φ_*pair*_(*t*, *m*) = Φ_*tar*_(*t*) ⊗ Φ_*mol*_(*m*),

that is, a (*n*_*tar *_× *n*_*mol*_)-dimensional vector whose entries are products of the form $\Phi_{tar}^{i}\left(t\right)\times \Phi_{mol}^{j}\left(m\right)$, for 1 ≤ *i *≤ *n*_*tar *_and 1 ≤ *j *≤ *n*_*mol*_. A SVM is then used as an inference engine, to estimate a linear function *f*(*t*, *m*) in the vector space of target/molecule pairs, that takes positive values for interacting pairs and negative values for non-interacting ones.

The main motivation for using the tensor product (1) is that it provides a systematic way to encode correlations between small molecule and target features. For example, in the case of binary descriptors, the product of two features is 1 if both the molecule and the target descriptors are 1, and zero otherwise, which amounts to encode the simultaneous presence of particular features of the molecule and of the target that may be important for the formation of a complex. A potential issue with this approach, however, is that the size of the vector representation *n*_*tar *_× *n*_*mol *_for a pair may be prohibitively large for practical computation and manipulation. For example, using a vector of molecular descriptors of size 1024 for molecules, and representing a protein by the vector of counts of all 2-mers of amino-acids in its sequence (*d*_*t *_= 20 × 20 = 400) results in more than 400 k dimensions for the representation of a pair. As pointed out by [27], this computational obstacle can however be overcome when a SVM is used to train the linear classifier, thanks to a trick often referred to as the *kernel trick*. Indeed, a SVM does not necessarily need the explicit computation of the vectors representing the complexes in the training set to train a model. What it needs, instead, is the inner products between these vectors, and a classical property of tensor products is that the inner product between two tensor products Φ_*pair*_(*t*, *m*) and Φ_*pair*_(*t'*, *m'*) is the product of the inner product between Φ_*tar*_(*t*) and Φ_*tar*_(*t'*), on the one hand, and the inner product between Φ_*mol*_(*m*) and Φ_*mol*_(*m'*), on the other hand. More formally, this property can be written as follows:

(2) $$\begin{array}{l} {\left(\Phi_{tar}\left(t\right)⊗\Phi_{mol}\left(m\right)\right)}^{⊤}\left(\Phi_{tar}\left(t^{\prime }\right)⊗\Phi_{mol}\left(m^{\prime }\right)\right)\\ =\Phi_{tar}{\left(t\right)}^{⊤}\Phi_{tar}\left(t^{\prime }\right)\times \Phi_{mol}{\left(m\right)}^{⊤}\Phi_{mol}\left(m^{\prime }\right), \end{array}$$

where *u *⊤ *v *= *u*_1_*v*_1 _+ ... + *u*_*d*_*v*_*d *_denotes the inner product between two *d*-dimensional vectors *u *and *v*. In other words, the SVM does not need to compute the *n*_*tar *_× *n*_*mol *_vectors to describe each pair, it only computes the respective inner products in the target and ligand spaces, before taking the product of both numbers.

This flexibility to manipulate molecule and target descriptors separately can moreover be combined with other tricks that sometimes allow to compute efficiently the inner products in the target and ligand spaces, respectively. Many such inner products, also called *kernels*, have been developed recently both in computational biology [37] and chemistry [38-40], and can be easily combined within the chemogenomics framework as follows: if two kernels for molecules and targets are given as:

(3) $$\begin{array}{c} K_{mol}\left(m,m^{\prime }\right)=\Phi_{mol}{\left(m\right)}^{⊤}\Phi_{mol}\left(m^{\prime }\right),\\ K_{tar}\left(t,t^{\prime }\right)=\Phi_{tar}{\left(t\right)}^{⊤}\Phi_{tar}\left(t^{\prime }\right), \end{array}$$

then we obtain the inner product between tensor products, *i.e.*, the kernel between pairs, by:

(4) *K*((*t*, *m*), (*t*', *m*')) = *K*_*tar*_(*t*, *t*') × *K*_*mol*_(*m*, *m*').

In summary, as soon as two vectors of descriptors or kernels *K*_*mol *_and *K*_*tar *_are chosen, we can solve the *in silico *chemogenomics problem with an SVM using the product kernel (4) between pairs. The particular descriptors or kernels used should ideally encode properties related to the ability of similar molecules to bind similar targets or ligands respectively.

In the next two subsections, we present different possible choices of descriptors – or kernels – for small molecules and GPCRs, respectively.

### Descriptors for small molecules

The problem of explicitly representing and storing small molecules as finite-dimensional vectors has a long history in chemoinformatics, and a multitude of molecular descriptors have been proposed [41]. These descriptors include in particular physicochemical properties of the molecules, such as its solubility or logP, descriptors derived from the 2D structure of the molecule, such as fragment counts or structural fingerprints, or descriptors extracted from the 3D structure [42]. Each classical fingerprint vector and vector representation of molecules define an explicit "chemical space" in which each molecule is represented by a finite-dimensional vector, and these vector representations can obviously be used as such to define kernels between molecules [43]. Alternatively, some authors have recently proposed some kernels that generalize some of these sets of descriptors and correspond to inner products between large- or even infinite-dimensional vectors of descriptors. These descriptors encode, for example, the counts of an infinite number of walks on the graph describing the 2D structure of the molecules [39,40,44], or various features extracted from the 3D structures [43,45].

In this study we select two existing kernels, encoding respectively 2D and 3D structural information of the small molecules:

• *The 2D Tanimoto kernel*. Our first set of descriptors is meant to characterize the 2D structure of the molecules. For a small molecule *m*, we define the vector Φ_*mol*_(*m*) as the binary vector whose bits indicate the presence or absence of all linear graph of length *u *or less as subgraphs of the 2D structure of *l*. We chose *u *= 8 in our experiment, *i.e.*, characterize the molecules by the occurrences of linear subgraphs of length 8 or less, a value previously observed to give good results in several virtual screening tasks [40]. Moreover, instead of directly taking the inner product between vectors as in (3), we use the Tanimoto kernel:

(5) $$\begin{array}{l} K_{ligand}\left(l,l^{\prime }\right)\\ =\frac{\Phi_{lig}{\left(l\right)}^{⊤}\Phi_{lig}\left(l^{\prime }\right)}{\Phi_{lig}{\left(l\right)}^{2}+\Phi_{lig}{\left(l^{\prime }\right)}^{2}-\Phi_{lig}{\left(l\right)}^{⊤}\Phi_{lig}\left(l^{\prime }\right)}, \end{array}$$

which was proven to be a valid inner product by [46], giving very competitive results on a variety of QSAR or toxicity prediction experiments.

• *3D pharmacophore kernel *While 2D structures are known to be very competitive in ligand-based virtual screening for identification of molecules presenting some given chemical, physical or biological properties [43], we reasoned that the protein-ligand recognition process takes place in the 3D space. Thus, we decided to test descriptors representing the presence of potential 3-point pharmacophores. For this, we used the 3D pharmacophore kernel proposed by [45], that generalizes 3D pharmacophore fingerprint descriptors. This approach requires the choice of a 3D conformer for each molecule, in a context where there exists a large number of methods for exploring the conformation space, and where we lack significant data for bound ligands in GPCR structures. Therefore, we chose to build a 3D version of the ligand database in which molecules are represented in the conformation proposed by the Omega program (OpenEye Scientific Software), because it performs rapid systematic conformer search, and has been showed to present good performances for retrieving bioactive conformations [47]. For each of the 2446 retained ligands, the conformer was generated using the standard Omega parameters, except for a 1 Å RMSD clustering of the conformers, instead of the 0.8 default value. Partial charges were calculated for all atoms using the molcharge program (OpenEye Scientific Software) with standard parameters. This ligand database was then used to calculate a 3D pharmacophore kernel for molecules [45].

We used the freely and publicly available *ChemCPP *(available at ) software to compute the 2D and 3D pharmacophore kernel.

### Descriptors for GPCRs

SVM and kernel methods are also widely used in bioinformatics [37], and a variety of approaches have been proposed to design kernels between proteins, ranging from kernels based on the amino-acid sequence of a protein [48-54] to kernels based on the 3D structures of proteins [55-57] or on the pattern of occurrences of proteins in multiple sequenced genomes [58]. These kernels have been used in conjunction with SVM or other kernel methods for various tasks related to structural or functional classification of proteins. While any of these kernels can theoretically be used as a GPCR kernel in (4), we investigate in this paper a restricted list of specific kernels described below, aimed at illustrating the flexibility of our framework and test various hypothesis.

• The *Dirac *kernel between two targets *t*, *t' *is:

(6) $$K_{Dirac}\left(t,t^{\prime }\right)=\{\begin{array}{ll} 1 & \text{if }t=t^{\prime },\\ 0 & \text{otherwise}\text{.} \end{array}$$

This basic kernel simply represents different targets as orthonormal vectors. From (4) we see that orthogonality between two proteins *t *and *t' *implies orthogonality between all pairs (*l*, *t*) and (*l'*, *t'*) for any two small molecules *c *and *c'*. This means that a linear classifier for pairs (*l*, *t*) with this kernel decomposes as a set of independent linear classifiers for interactions between molecules and each target protein, which are trained without sharing any information of known ligands between different targets. In other words, using Dirac kernel for proteins amounts to performing classical learning independently for each target, which is our baseline approach.

• The *multitask *kernel between two targets *t*, *t' *is defined as:

*K*_*multitask*_(*t*, *t'*) = 1 + *K*_*Dirac*_(*t*, *t'*).

This kernel, originally proposed in the context of multitask learning [59], removes the orthogonality of different proteins to allow sharing of information. As explained in [59], plugging *K*_*multitask*_in (4) amounts to decomposing the linear function used to predict interactions as a sum of a linear function common to all GPCRs and of a linear function specific to each GPCR:

$$f\left(l,t\right)=w^{⊤}\Phi \left(l,t\right)=w_{general}^{⊤}\Phi_{lig}\left(l\right)+w_{t}^{⊤}\Phi_{lig}\left(l\right).$$

A consequence is that only data related to the the target *t *are used to estimate the specific vector *w*_*t*_, while all data are used to estimate the common vector *w*_*general*_. In our framework this classifier is therefore the combination of a target-specific part accounting for target-specific properties of the ligands and a global part accounting for general properties of the ligands across the targets. The latter term allows to share information during the learning process, while the former ensures that specificities of the ligands for each target are not lost.

• The *hierarchy *kernel. Alternatively we could propose a new kernel aimed at encoding the similarity of proteins with respect to the ligands they bind. In the GLIDA database indeed, GPCRs are grouped into 4 classes based on sequence homology and functional similarity: the *rhodopsin *family (class A), the *secretin *family (class B), the *metabotropic *family (class C) and some smaller classes containing other GPCRs. The GLIDA database further subdivides each class of targets by type of ligands, for example amine or peptide receptors or more specific families of ligands. This also defines a natural hierarchy that can be used to compare GPCRs.

The hierarchy kernel between two GPCRs was therefore defined as the number of common ancestors in the corresponding hierarchy plus one, that is,

*K*_*hierarchy*_(*t*, *t*') = ⟨Φ_*h*_(*t*), Φ_*h*_(*t'*)⟩,

where Φ_*h*_(*t*) contains as many features as there are nodes in the hierarchy, each being set to 1 if the corresponding node is part of *t*'s hierarchy and 0 otherwise, plus one feature constantly set to one that accounts for the "plus one" term of the kernel.

**Figure 1:**
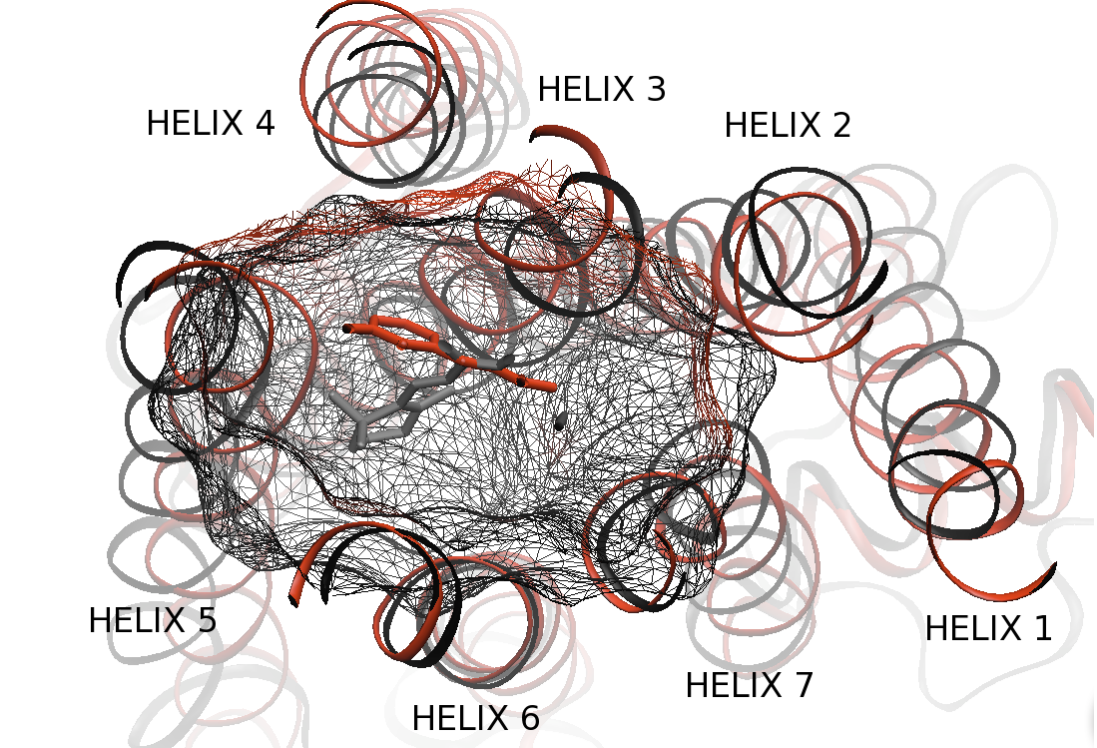
**Binding pocket**. Representation of the binding pocket of *β*_2_-adrenergic receptor (in red) and bovine Rhodopsin (in black) viewed from the extracellular surface. On the center of the pocket, 3-(isopropylamino)propan-2-ol and cis-retinal have been represented to show the size and the position of the pocket around each ligand. Figure drawn with VMD [79].

**Figure 2:**
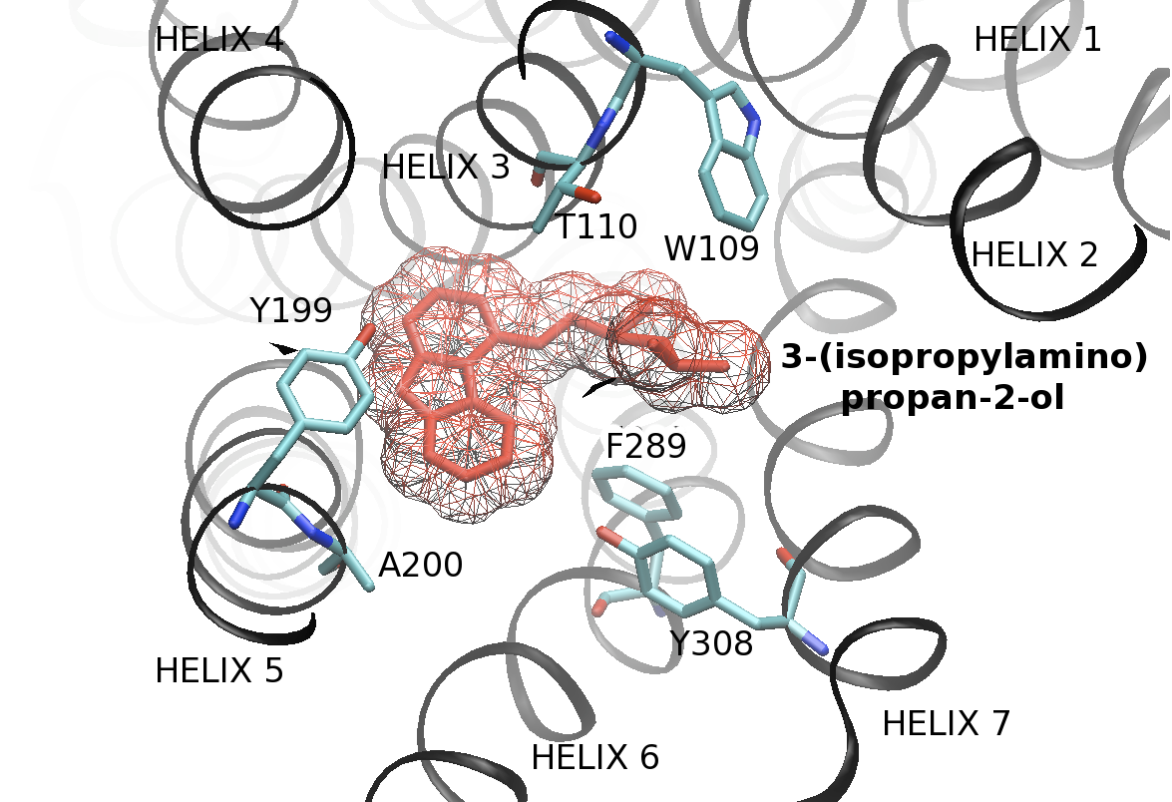
**3-(isopropylamino)propan-2-ol and the protein environment of *β*_2_-adrenergic receptor as viewed from the extracellular surface**. 3-(isopropylamino)propan-2-ol and the protein environment of *β*_2_-adrenergic receptor as viewed from the extracellular surface. Amino acid side chains are represented for 6 of the 31 residues (in cyan, blue and red) of the binding pocket motif. Transmembrane helix and 3-(isopropylamino)propan-2-ol are colored in black and red respectively. Figure drawn with VMD [79].

• The *binding pocket *kernel. Because the protein-ligand recognition process occurs in 3D space in a pocket involving a limited number of residues, we tried to describe the GPCR space using a representation of this pocket. The difficulty resides in the fact that although the GPCR sequences are known, the residues forming this pocket are *a priori *unknown. However, mutagenesis data showed that the transmembrane binding site is situated in a similar region for all GPCRs [60], and this information was confirmed by the two available X-ray structures. In order to identify residues potentially involved in the binding pocket of GPCRs of unknown structure studied in this work, we proceeded in several steps, somewhat similarly to [61]. (a) The two known structures, PDB entries 1U19 and 2RH1[62,63], were superimposed using the STAMP algorithm [64]. Although retinal is an inverse agonist and form a covalent bond with Rhodopsin, while carazolol is an agonist and binds non-covalently, root mean square deviation between these two complexed structures is only of 1.6 Å in the transmembrane helices [65]. In the superimposed structures, the retinal and 3-(isopropylamino)propan-2-ol ligands are localized in the same region of the transmembrane space, which is in agreement with global conservation of binding pockets, as shown on Figure 1. (b) The structural alignment of bovine rhodopsin and of human *β*_2_-adrenergic receptor was used to generate a sequence alignment of these two proteins. (c) For both structures, in order to identify residues potentially involved in stabilizing interactions with the ligand (residues of the pocket), we selected residues that presented at least one atom situated at less than 6 Å from at least one atom of the ligand. Figure 2 shows that these two pockets clearly overlap, as expected. (d) Residues of the two pockets (as defined in (c)) were labeled in this structural sequence alignment. These residues were found to form small sequence clusters that were in correspondence in this alignment. These clusters were situated mainly in the apical region of transmembrane segments and included a few extracellular residues. Indeed, it has been previously demonstrated that extracellular loops can play a role in ligand binding together with transmembrane regions [66]. (e) All studied GPCR sequences, including bovine rhodopsin and human *β*_2_-adrenergic receptor were aligned using CLUSTALW [67] with Blosum matrices [68]. Sequences which could not be correctly aligned (i.e. with important gaps in the transmembrane regions) were discarded in order to only keep comparable sequences. We then checked that conserved residues according to [69] of the transmembrane helices were correctly aligned, and local misalignments were corrected. In addition, the structural alignment of bovine rhodopsin and human *β*_2_-adrenergic receptor, and known conserved positions were used to locally correct misalignments. For each protein, residues in correspondence in this alignment with a residue of the binding pocket (as defined above) of either bovine rhodopsin or human *β*_2_-adrenergic receptor were retained. This lead to a different number of residues per protein, because of sequence variability. For example, in extracellular regions, some residues from bovine rhodopsin or human *β*_2_-adrenergic receptor had a corresponding residue in some sequences but not in others. In order to provide a homogeneous description of the binding pocket for all GPCRs, in the list of residues initially retained for each protein, only residues situated at positions where no gaps were found in any of the GPCRs were kept. (f) Each protein was then represented by a vector whose elements corresponded to a potentially conserved pocket. This description, although appearing as a linear vector filled with amino acid residues [see Additional file 1], implicitly codes for a 3D information on the receptor pocket, as illustrated in Figure 2. These vectors were then used to build a kernel that allows comparison of binding pockets. The classical way to represent motifs of constant length as fixed length vectors is to encode the letter at each position by a 20-dimensional binary vector indicating which amino acid is present, resulting in a 180-dimensional vector representations. In terms of kernel, the inner product between two binding pocket motifs in this representation is simply the number of letters they have in common at the same positions:

$$K_{pb}\left(x,x^{\prime }\right)=\sum_{i=1}^{l}\delta \left(x\left[i\right],x^{\prime }\left[i\right]\right),$$

where *l *is the length of the binding pocket motifs (31 in our case), *x*[*i*] is the *i*-th residue in x and *δ *(*x*[*i*], *x'*[*i*]) is 1 if *x*[*i*] = *x'*[*i*], 0 otherwise. This is the baseline pocket binding kernel. Alternatively, using a polynomial kernel of degree *p *over the baseline kernel is equivalent, in terms of feature space, to encoding *p*-order interactions between amino acids at different positions. In order to assess the relevance of such non-linear extensions we tested this polynomial pocket binding kernel,

*K*_*ppb*_(*x*, *x*') = (*K*_*pb*_(*x*, *x*') + 1)^*p*^.

We only used a degree *p *= 2, although a more careful choice of this parameter could further improve the performances.

## Results

We ran two different sets of experiments on this dataset in order to illustrate two important points. In a first set of experiments, for each GPCR, we 5-folded the data available, *i.e.*, the line of the interaction matrix corresponding to this GPCR. The classifier was trained with four folds and the whole data from the other GPCRs, *i.e.*, all other lines of the interaction matrix. The prediction accuracy for the GPCR under study was then tested on the remaining fold. The goal of these first experiments was to evaluate if using data from other GPCRs improved the prediction accuracy for a given GPCR. In a second set of experiments, for each GPCR we ignored ligand data available for this particular GPCR, we trained a classifier on the whole data from the other GPCRs, and tested on the data of the considered GPCR. The goal was to assess how efficient our chemogenomics approach would be to predict the ligands of orphan GPCRs. In both experiments, the *C *parameter of the SVM was selected by internal cross validation on the training set among 2^*i*^, *i *∈ {-8, -7,..., 5, 6}. The data and source code (under GPL license) are publicly available [see Additional file 2].

For the first experiment, since learning an SVM with only one training point does not really make sense and can lead to "anti-learning" less than 0.5 performances, we set all results *r *involving the Dirac GPCR kernel on GPCRs with only 1 known ligand to max(*r*, 0.5). This is to avoid any artefactual penalization of the Dirac approach and make sure that we measure the actual improvement brought by sharing information across GPCRs.

**Figure 3:**
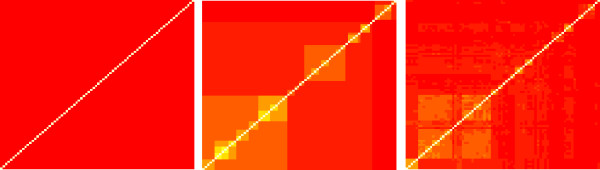
**GPCR kernel Gram matrices**. GPCR kernel Gram matrices (*K*_*tar*_) for the GLIDA GPCR data with multitask, hierarchy and binding pocket kernels.

Table 1 shows the results of the first experiments with all the ligand and GPCR kernel combinations. For all the ligand kernels, one observes an improvement between the individual approach (Dirac GPCR kernel, 86.2%) and the baseline multitask approach (multitask GPCR kernel, 88.8%). The latter kernel is merely modeling the fact that each GPCR is uniformly similar to all other GPCRs, and twice more similar to itself. It does not use any prior information on the GPCRs, and yet, using it improves the global performance with respect to individual learning. Using more informative GPCR kernels further improves the prediction accuracy. In particular, the hierarchy kernel add more than 4.5% of precision with respect to naive multitask approach. All the other informative GPCR kernels also improve the performance. The polynomial binding pocket kernel is almost as efficient as the hierarchy kernel, which is an interesting result. Indeed, one could fear that using the hierarchy kernel, for the construction of which some knowledge of the ligands may have been used, could have introduced bias in the results. Such bias is certainly absent in the binding pocket kernel. The fact that the same performance can be reached with kernels based on the mere sequence of GPCRs' pockets is therefore an important result. Figure 3 shows three of the GPCR kernels. The baseline multitask is shown as a comparison. Interestingly, many of the subgroups defined in the hierarchy can be found in the binding pocket kernel, that is, they are retrieved from the simple information of the binding pocket sequence.

**Table 1:** Prediction accuracy for the first experiment with various ligand and target kernels

*K*_*tar*_\*K*_*lig*_	2D Tanimoto	3D pharmacophore
Dirac	86.2 ± 1.9	84.4 ± 2.0
multitask	88.8 ± 1.9	85.0 ± 2.3
hierarchy	93.1 ± 1.3	88.5 ± 2.0
binding pocket	90.3 ± 1.9	87.1 ± 2.3
poly binding pocket	92.1 ± 1.5	87.4 ± 2.2

The 3D kernel for the ligands, on the other hand, did not perform as well as the 2D kernel. This can be either explained by the fact the the pharmacophore kernel is not suited to this problem, or by the fact that choosing the conformer of the ligand is not a trivial task. This point is discussed below.

**Figure 4:**
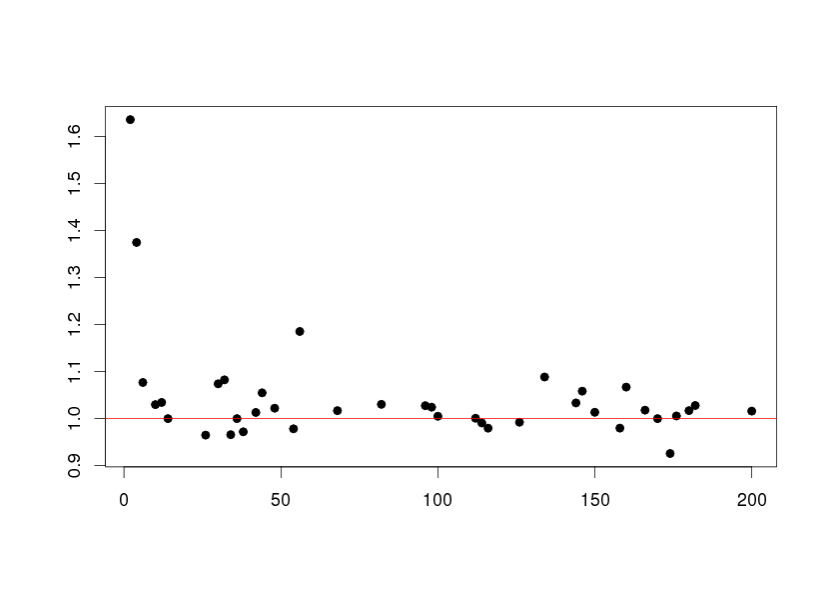
**Improvement of the chemogenomics approach**. Improvement (as a performance ratio) of the hierarchy GPCR kernel against the Dirac GPCR kernel as a function of the number of training samples available. Restricted to [2 – 200] samples for the sake of readability.

**Figure 5:**
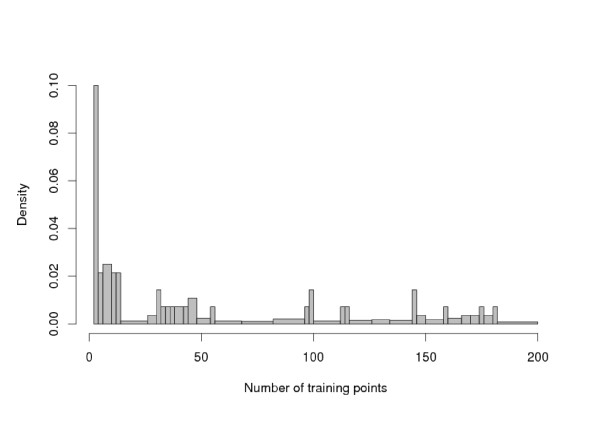
**Distribution of the number of training points for a GPCR**. Distribution of the number of training points for a GPCR. Restricted to [2 – 200] samples for the sake of readability.

Figure 4 illustrates how the improvement brought by the chemogenomics approach varies with the number of available training points. As one could have expected, the strongest improvement is observed for the GPCRs with few (less than 20) training points (*i.e.*, less than 10 known ligands since for each known ligand an artificial non-ligand was generated). When more training points become available, the improvement is less important, and sharing the information across the GPCRs can even degrade the performances. This is an important point, first because, as showed on Figure 5, many GPCRs have few known ligands (in particular, 11 of them have only two training points), and second because it shows that when enough training points are available, individual learning will probably perform as well as or better than our chemogenomics approach.

Our second experiment intends to assess how our chemogenomics approach can perform when predicting ligands for orphan GPCRs, *i.e.*, with no training data available for the GPCR of interest. Table 2 shows that in this setting, individual learning performs random prediction. Naive multitask approach provides modest improvement of the performance, but informative kernels such as hierarchical and binding pocket kernels achieve 77.4% and 78.1% of precision respectively, that is, almost 30% better than the random approach one would get when no data is available. Here again, the fact that the binding pocket kernel that only uses the sequence of the receptor pocket performs as well as the hierarchy-based kernel is encouraging. It suggests that given a receptor for which nothing is known except its sequence, it is possible to make reasonable ligand predictions.

**Table 2:** Prediction accuracy for the second experiment with various ligand and target kernels

*K*_*tar*_\*K*_*lig*_	2D Tanimoto	3D pharmacophore
Dirac	50.0 ± 0.0	50.0 ± 0.0
multitask	56.8 ± 2.5	58.2 ± 2.2
hierarchy	77.4 ± 2.4	76.2 ± 2.2
binding pocket	78.1 ± 2.3	76.6 ± 2.2
poly binding pocket	76.4 ± 2.4	74.9 ± 2.3

## Discussion

Our results demonstrate that chemogenomic approaches outperform individual approach, in particular in cases where very limited or no ligand information is available, as shown in Table 2 and Figure 4. In the case of well studied GPCRs, more classical ligand-based methods (QSAR) may be better suited to predict new strong binders from a large number of known ligands, as shown in Figure 4. Consistent with this observation, Tables 3 and 4 show that in the two types of experiments, the improvement is observed for all subfamilies of GPCRs retained in this study. This is an interesting result since most of published virtual screening studies on GPCRs were applied to class A GPCRs.

**Table 3:** Prediction accuracy by GPCR family for the first experiment

Family\*K*_*tar*_	Dirac	multitask	hierarchy	BP	PBP
Rhodopsin peptide receptors (18)	73.7	80.0	85.8	83.8	83.7
Rhodopsin amine receptors (35)	91.1	92.1	94.0	93.9	94.1
Rhodospin other receptors (17)	83.6	88.0	95.7	95.9	95.9
Metabotropic glutamate family (9)	73.1	93.5	98.9	83.3	93.3
Secretin family (1)	50.0	100.0	100.0	50.0	100.0

Mean prediction accuracy for each GPCR family for the first experiment with the 2D Tanimoto ligand kernel and various target kernels. The numbers in bracket are the numbers of receptors considered in the experiment for each family. BP is the binding pocket kernel and PBP the poly binding pocket kernel.

**Table 4:** Prediction accuracy by GPCR family for the second experiment

Family\*K*_*tar*_	Dirac	multitask	hierarchy	BP	PBP
Rhodopsin peptide receptors (18)	50.0	50.6	66.7	74.0	65.3
Rhodopsin amine receptors (35)	50.0	56.0	73.7	74.0	73.1
Rhodospin other receptors (17)	50.0	50.2	86.5	87.6	85.5
Metabotropic glutamate family (9)	50.0	79.7	93.9	87.2	91.3
Secretin family (1)	50.0	100.0	100.0	50.0	100.0

Mean prediction accuracy for each GPCR family for the second experiment with the 2D Tanimoto ligand kernel and various target kernels. The numbers in bracket are the numbers of receptors considered in the experiment for each family. BP is the binding pocket kernel and PBP the poly binding pocket kernel.

Since our chemogenomic approach is a ligand-based approach, it would probably be interesting to use it in combination with docking. Indeed, although prior known ligands can help tuning docking procedures to the receptor under study, it can in principle be used with little or no ligand information. When more experimental 3D structures become available for GPCRs in the future, this will help building reliable models for a wider range of GPCRs that would be suitable for docking studies. Joint use of ligand-based chemogenomic and docking would certainly improve predictions.

We chose to use a binary descriptor for the receptor-ligand interaction, while QSAR or docking methods usually try to rank molecules according to their predicted affinity for the receptor. However, affinity prediction is still a subject of research at the level of a single receptor, at least when using methods whose calculation times are compatible with the screening of large molecular databanks. In this context, we feel that in chemogenomic approaches, where information is shared between different proteins, such quantitative prediction is even more challenging. This led us to retain the binary binding and non-binding descriptors, although it would formally have been straightforward to use a regression algorithm instead of a classification one to make quantitative predictions.

It is not always easy to compare the performances of a new method to other existing methods, and particularly in the case of GPCRs. Indeed, at least to our knowledge, there is up to now no public complete data from previous screening studies available as a benchmark to compare different screening methods on the same data. This urged us to give public access to the ligand and receptor databases used in this study, to the detailed experimental protocol of the study, and to the predictions made by our chemogenomic approach for each GPCR [see Additional files 3, 4] (summarized by GPCR family in Table 3 and Table 4). This provides a benchmark which we hope will contribute to a fair evaluation of different methods and trigger new developments. This benchmark could be used to compare predictions made by other methods. Our approach boils down to the application of well-known machine learning methods in the constructed chemogenomics space. We used a systematic way to build such a space by combining a given representation of the ligands with a given representation of the GPCRs into a binding-prediction-oriented GPCR-ligand couple representation. This allows to use any ligand or GPCR descriptor or kernel existing in the chemoinformatics or bioinformatics literature, or new ones containing other prior information as we tried to propose in this paper. Our experiments showed that the choice of the descriptors was crucial for the prediction, and more sophisticated features for either the ligands or the GPCRs could probably further improve the performances. Among these features, improvements in the 3D ligand descriptors could probably be obtained. Indeed, 3D pharmacophore kernels did not always reach the performance of 2D kernels for the ligands. This is apparently in contradiction with the idea that protein-ligand interaction is a process occurring in the 3D space, and with previous work in our group [45]. Different explanations can be proposed. First, it is possible that the bioactive conformation was not correctly predicted for all molecules used in this study. For the two ligands for which it was known, *i.e.*, retinal and 3-(isopropylamino)propan-2-ol from PDB entries 1U19 and 2RH1 respectively, we found that the predicted conformation, using the same method as for all other molecules, was very close to the experimental conformation, with RMSD values of less than 1 Å. However, in absence of any other information on bound ligand conformations, it is not possible to rule out the possibility that for other molecules, the prediction was not correct. Although more complete conformational space exploration for all ligands was clearly out of the scope of this paper and would be a study by itself, work in this direction could improve the method. In particular, since 2D ligand-based methods are not easily suitable to make predictions outside of the molecular scaffolds for which information is known, ligand-based methods using 3D description are of particular interest, because they are expected to allow better predictions on molecules presenting diverse molecular patterns. Synergy between our method and docking would provide a means for the choice of a conformer. The principle could be to build homology models for the GPCRs, dock the molecular database in the modeled binding pockets, and derive a 3D database using, for each molecule, the conformer associated to the best docking solution. However, conformer generation and selection is a major drawback of using 3D descriptors, especially in the case of large ligands with many free torsion angles.

Various evidence suggest that, within a common global architecture, a generic binding pocket mainly involving transmembrane regions hosts agonists, antagonists and allosteric modulators. In order to identify this pocket automatically, other studies report the use of sequence alignment and the prediction of transmembrane helices. [60] detected hypervariable positions in transmembrane helices for identification of residues forming the binding pocket, although some positions were more conserved. Indeed, conserved residues are probably important for structural stabilization of the pocket, while variable positions are involved in ligand binding, in order to accommodate the wide spectrum of molecules that are GPCR substrates. Analyzing the positions of variable positions, these authors proposed potential binding pockets for GPCRs, and found that the corresponding residues were frequently in the GRAP mutant database for GPCRs [70]. Interestingly, they pointed that residues at hypervariable positions were found within a distance of 6 Å from retinal in the rhodopsin X-Ray structure, which is also a classical distance cutoff above which it is admitted that protein-ligand interactions become negligible. Therefore, this inspired the simple and automatic method used in the present work for extracting GPCRs potential binding pockets, and our results are in good agreement with this study. It is also important to note that GPCRs are known to exist in dynamic equilibrium between inactive- and several active-state conformations [71], and different ligands sometimes trigger distinct conformational changes and stabilize different receptor conformations [72]. Taking into account receptor plasticity constitutes in itself a research domain in docking. Its use is of particular interest for screening GPCR homology models since residue positions are not exactly known. Therefore flexible docking procedures have been proposed and applied on GPCR proteins [9,73]. Moreover, a modeling method has been proposed to get insights on transmembrane bundle plasticity [74]. In our case, receptor flexibility might influence the definition of the binding pocket, since it initially relies on the identification of residues in the two reference structures (1U19 and 2RH1) that present at least one atom situated at less than 6 Å of the ligand. Therefore, we made the implicit hypothesis that receptor conformational changes upon ligand binding does not drastically affect this list of residues. When more structures become available in this family of proteins, a better appreciation of such conformational rearrangements will be possible, which could be taken into account in the binding pocket definition and could help to improve the method. [70] found that hierarchical tree representations of GPCR subfamilies calculated with full-length GPCR sequences or with only binding pocket residues were similar, and that locally, the latter was in better agreement with functional data although their binding pocket included only 35 residues. This result is also in good agreement with our finding that the hierarchy kernel based on full length sequence (from GLIDA) and the kernel based on the binding pocket provided very similar performances. As mentioned in the Results section, it is however important to note that the kernels based on the binding pocket were built without any ligand information that could lead to some bias and artificially better performance.

## Conclusion

We showed how sharing information across the GPCRs by considering a chemogenomics space of the GPCR-ligand interaction pairs could improve the prediction performances, with respect to the single receptor approach. In addition, we showed that using such a representation, it was possible to make reasonable predictions even when all known ligands were ignored for a given GPCR, that is, to predict ligands for orphan GPCRs. Our results demonstrate that chemogenomic approaches is particularly suited to cases where very limited or no ligand information is available, as shown in Table 2.

This chemogenomics approach is related to ligand-based approaches. However, sharing information among different GPCRs allows, in this case, to perform prediction on orphan GPCRs, which is also possible using target-based methods. Nevertheless, the latter are limited by the number of known receptor structures and the difficulty to apply such methods on homology models.

Interesting developments of this method could include application to other important drug target families, like enzymes or ion channels [75], for which most of the descriptors used for the GPCRs in this paper could directly be transposed, and other, more specific ones could be designed. From a methodological point of view, many recent developments in multitask learning [76-78] could be applied to generalize this chemogenomics approach using, for example, other regularization methods.

## Authors' contributions

LJ, with the help and under the supervision of JPV, developed and implemented all the classification methods presented in the paper, and ran the experiments. BH and VS designed the benchmark and developed the binding pocket kernel and 3D ligand kernel. All authors contributed to writing the text, read and approved the final manuscript.

## Supplementary Material

Additional file 1 Aligned receptor pocket residues. Residues of 5-hydroxytryptamine 5A receptor, Adenosine A2b receptor, Gamma-aminobutyric acid type B receptor and Relaxin 3 receptor 2 (shown as examples) aligned with *β*_2_-adrenergic receptor binding site amino acids. The binding pocket motif of *β*_2_-adrenergic receptor has been used as reference to determine residues involved in the formation of the binding site of the 79 other GPCRs. Bold columns correspond to the residues shown on Figure 2. Click here for file

Additional file 2 Source and data. Source code (under GPL license) and benchmark used in the experiments in a compressed archive checker.tgz. Click here for file

Additional file 3 Prediction accuracy by GPCR for the first experiment. Mean prediction accuracy for each GPCR for the first experiment with the 2D Tanimoto ligand kernel and various target kernels. The GPCR identifiers are the GLIDA references. The numbers in bracket are the numbers ligands considered in the experiment for each GPCR. BP is the binding pocket kernel and PBP the poly binding pocket kernel. Click here for file

Additional file 4 Prediction accuracy by GPCR for the second experiment. Mean prediction accuracy for each GPCR for the second experiment with the 2D Tanimoto ligand kernel and various target kernels. The GPCR identifiers are the GLIDA references. The numbers in bracket are the numbers ligands considered in the experiment for each GPCR. BP is the binding pocket kernel and PBP the poly binding pocket kernel. Click here for file
