# Supplementary material for: Virtual screening of GPCRs: An in silico chemogenomics approach
Source: BMC Bioinformatics. 2008 Sep 6;9:363. doi: 10.1186/1471-2105-9-363 (PMC2553090; doi:10.1186/1471-2105-9-363)
Supplement: Additional file 1 — Aligned receptor pocket residues. Residues of 5-hydroxytryptamine 5A receptor, Adenosine A2b receptor, Gamma-aminobutyric acid type B receptor and Relaxin 3 receptor 2 (shown as examples) aligned with β2-adrenergic receptor binding site amino acids. The binding pocket motif of β2-adrenergic receptor has been used as reference to determine residues involved in the formation of the binding site of the 79 other GPCRs. Bold columns correspond to the residues shown on Figure 2. [file 1471-2105-9-363-S1.pdf]

positions on β_2-adrenergic receptor	82	**109**	**110**	113	114	115	116	117	118	121	175	183	195	**199**	**200**
β_2-adrenergic receptor	M	**W**	**T**	D	V	L	C	V	T	I	R	N	T	**Y**	**A**
5-hydroxytryptamine 5A receptor	V	**W**	**I**	D	V	L	C	C	T	I	I	E	S	**Y**	**A**
Adenosine A2b receptor	V	**L**	**A**	V	L	V	L	T	Q	I	I	K	K	**M**	**V**
Gamma-aminobutyric acid type B receptor	E	**D**	**E**	E	A	V	E	G	H	T	L	G	S	**F**	**D**
Relaxin 3 receptor 2	L	**V**	**L**	T	V	L	N	V	Y	I	V	G	L	**Y**	**Q**

positions on β_2-adrenergic receptor	203	204	207	208	212	282	286	**289**	290	293	**308**	311	312	313	315	316
β_2-adrenergic receptor	S	S	S	F	L	F	W	**F**	F	N	**Y**	L	N	W	G	Y
5-hydroxytryptamine 5A receptor	S	T	A	F	L	F	W	**F**	F	E	**K**	F	L	W	G	Y
Adenosine A2b receptor	N	F	C	V	L	F	W	**V**	H	N	**M**	A	I	L	S	H
Gamma-aminobutyric acid type B receptor	G	S	A	W	E	F	L	**Y**	H	R	**L**	T	V	G	L	V
Relaxin 3 receptor 2	R	V	A	F	L	F	W	**N**	H	T	**F**	T	T	C	A	H
