# Supplementary material for: Virtual screening of GPCRs: An in silico chemogenomics approach
Source: BMC Bioinformatics. 2008 Sep 6;9:363. doi: 10.1186/1471-2105-9-363 (PMC2553090; doi:10.1186/1471-2105-9-363)
Supplement: Additional file 3 — Prediction accuracy by GPCR for the first experiment. Mean prediction accuracy for each GPCR for the first experiment with the 2D Tanimoto ligand kernel and various target kernels. The GPCR identifiers are the GLIDA references. The numbers in bracket are the numbers ligands considered in the experiment for each GPCR. BP is the binding pocket kernel and PBP the poly binding pocket kernel. [file 1471-2105-9-363-S3.pdf]

GPCR \ K_{tar}	Dirac	multitask	hierarchy	BP	PBP
Rhodopsin peptide receptors					
AG2R(5)	100.0 ± 0.0	100.0 ± 0.0	100.0 ± 0.0	100.0 ± 0.0	100.0 ± 0.0
CCKAR(6)	100.0 ± 0.0	100.0 ± 0.0	100.0 ± 0.0	100.0 ± 0.0	100.0 ± 0.0
CML2(1)	50.0 ± 0.0	50.0 ± 35.4	100.0 ± 0.0	50.0 ± 35.4	50.0 ± 35.4
CXCR3(1)	50.0 ± 35.4	0.0 ± 0.0	50.0 ± 35.4	50.0 ± 35.4	50.0 ± 35.4
EDNRA(50)	100.0 ± 0.0	99.0 ± 0.9	100.0 ± 0.0	100.0 ± 0.0	100.0 ± 0.0
EDNRB(48)	96.9 ± 1.1	91.8 ± 3.4	98.0 ± 1.1	99.0 ± 0.9	99.0 ± 0.9
GASR(2)	100.0 ± 0.0	75.0 ± 21.7	75.0 ± 21.7	75.0 ± 21.7	75.0 ± 21.7
GPR7(1)	50.0 ± 35.4	50.0 ± 35.4	50.0 ± 35.4	50.0 ± 35.4	50.0 ± 35.4
LSHR(4)	70.0 ± 11.0	70.0 ± 11.0	70.0 ± 11.0	70.0 ± 11.0	70.0 ± 11.0
NK1R(24)	92.0 ± 4.4	82.0 ± 5.2	86.0 ± 5.4	88.0 ± 3.3	86.0 ± 3.6
NK2R(1)	50.0 ± 35.4	100.0 ± 0.0	100.0 ± 0.0	100.0 ± 0.0	100.0 ± 0.0
NK3R(1)	50.0 ± 0.0	100.0 ± 0.0	100.0 ± 0.0	100.0 ± 0.0	100.0 ± 0.0
OPRD(27)	92.3 ± 1.7	86.7 ± 4.4	90.3 ± 4.9	90.3 ± 2.8	90.3 ± 2.8
OPRK(24)	96.0 ± 3.6	98.0 ± 1.8	98.0 ± 1.8	98.0 ± 1.8	98.0 ± 1.8
OPRM(21)	100.0 ± 0.0	97.5 ± 2.2	97.5 ± 2.2	97.5 ± 2.2	97.5 ± 2.2
OXYR(3)	90.0 ± 8.9	100.0 ± 0.0	90.0 ± 8.9	100.0 ± 0.0	100.0 ± 0.0
SSR1(3)	90.0 ± 8.9	90.0 ± 8.9	90.0 ± 8.9	90.0 ± 8.9	90.0 ± 8.9
CCR3(1)	50.0 ± 35.4	50.0 ± 35.4	50.0 ± 35.4	50.0 ± 35.4	50.0 ± 35.4
Rhodopsin amine receptors					
5HT1A(196)	91.6 ± 1.3	90.1 ± 2.2	88.8 ± 0.8	91.8 ± 1.5	90.8 ± 1.7
5HT1B(28)	82.7 ± 3.0	96.0 ± 3.6	98.0 ± 1.8	100.0 ± 0.0	100.0 ± 0.0
5HT1D(172)	93.3 ± 1.0	92.4 ± 0.9	92.7 ± 0.9	94.8 ± 0.7	94.8 ± 0.7
5HT1E(16)	87.5 ± 5.5	90.8 ± 3.4	96.7 ± 3.0	90.8 ± 3.4	90.8 ± 3.4
5HT1F(49)	86.7 ± 1.2	90.9 ± 0.8	88.8 ± 1.7	92.9 ± 1.1	91.7 ± 2.1
5HT2A(79)	94.9 ± 1.4	95.6 ± 1.4	93.0 ± 1.7	94.3 ± 1.7	94.9 ± 1.4
5HT2B(72)	81.2 ± 3.3	78.3 ± 2.9	83.9 ± 1.8	83.2 ± 2.0	83.2 ± 2.0
5HT2C(198)	88.6 ± 1.2	86.8 ± 1.2	89.4 ± 1.4	89.6 ± 0.8	90.1 ± 1.3
5HT4R(87)	92.5 ± 2.0	86.7 ± 2.5	85.7 ± 2.0	87.9 ± 2.1	89.0 ± 2.0
5HT5A(7)	80.0 ± 8.4	75.0 ± 10.0	75.0 ± 10.0	75.0 ± 10.0	75.0 ± 10.0
5HT6R(13)	95.0 ± 4.5	96.7 ± 3.0	91.7 ± 4.7	95.0 ± 4.5	100.0 ± 0.0
5HT7R(15)	90.0 ± 6.0	90.0 ± 3.7	96.7 ± 3.0	93.3 ± 3.7	93.3 ± 3.7
ACM1(527)	96.7 ± 0.6	94.3 ± 0.9	95.5 ± 1.0	96.1 ± 0.7	96.1 ± 0.8
ACM2(24)	82.0 ± 5.2	90.0 ± 2.8	92.0 ± 3.3	94.0 ± 3.6	92.0 ± 3.3
ACM3(58)	93.2 ± 2.6	90.5 ± 0.7	91.3 ± 1.3	96.4 ± 1.5	95.6 ± 1.3
ACM4(21)	90.0 ± 5.5	95.0 ± 2.7	95.0 ± 2.7	92.5 ± 2.7	95.0 ± 2.7
ACM5(16)	94.2 ± 3.2	94.2 ± 3.2	100.0 ± 0.0	100.0 ± 0.0	100.0 ± 0.0
ADA1A(80)	93.1 ± 2.1	98.8 ± 0.7	99.4 ± 0.6	98.1 ± 0.7	98.8 ± 0.7
ADA1B(67)	90.5 ± 3.7	95.7 ± 1.9	98.6 ± 0.8	97.0 ± 0.7	97.0 ± 0.7
ADA1D(73)	90.4 ± 2.4	96.0 ± 1.1	98.7 ± 0.7	98.0 ± 0.7	98.0 ± 0.7
ADA2A(234)	95.7 ± 0.5	96.8 ± 0.3	98.5 ± 0.2	98.5 ± 0.2	98.5 ± 0.2
ADA2B(224)	95.1 ± 1.2	95.5 ± 1.3	98.2 ± 0.7	98.2 ± 0.7	98.0 ± 0.7
ADA2C(225)	95.3 ± 0.4	96.4 ± 0.4	97.6 ± 0.4	97.6 ± 0.4	97.8 ± 0.3
ADRB1(50)	98.0 ± 1.1	97.0 ± 1.8	99.0 ± 0.9	99.0 ± 0.9	99.0 ± 0.9
ADRB2(48)	92.8 ± 1.9	95.9 ± 0.9	96.9 ± 1.1	98.0 ± 1.1	98.0 ± 1.1
ADRB3(57)	98.2 ± 1.0	95.5 ± 2.2	97.3 ± 1.6	97.3 ± 1.6	97.3 ± 1.6
DRD1(100)	93.5 ± 1.8	94.5 ± 1.5	95.0 ± 1.4	94.5 ± 1.3	94.5 ± 1.3
DRD2(106)	93.4 ± 0.8	92.9 ± 1.8	92.4 ± 1.6	91.5 ± 1.7	91.9 ± 1.9
DRD3(41)	86.7 ± 2.6	89.2 ± 3.1	89.3 ± 3.8	90.4 ± 3.2	91.5 ± 2.8
DRD4(143)	92.3 ± 0.8	92.7 ± 1.1	93.7 ± 1.3	93.7 ± 1.4	94.1 ± 1.3
DRD5(7)	95.0 ± 4.5	100.0 ± 0.0	100.0 ± 0.0	100.0 ± 0.0	100.0 ± 0.0
HRH1(19)	89.2 ± 4.3	92.5 ± 2.7	86.7 ± 0.7	92.5 ± 2.7	92.5 ± 2.7
HRH2(22)	91.0 ± 3.5	93.5 ± 3.7	96.0 ± 3.6	96.0 ± 3.6	96.0 ± 3.6
HRH3(88)	97.2 ± 0.8	96.1 ± 1.3	97.7 ± 0.9	97.7 ± 0.5	97.7 ± 0.5
HRH4(5)	80.0 ± 11.0	70.0 ± 17.9	100.0 ± 0.0	80.0 ± 11.0	80.0 ± 11.0

GPCR $\backslash K_{tar}$	Dirac	multitask	hierarchy	BP	PBP
		Rhodopsin other receptors			
AA1R(56)	96.4 ± 1.5	96.4 ± 0.8	96.4 ± 1.5	97.3 ± 1.0	97.3 ± 1.0
AA2AR(73)	96.0 ± 1.7	97.3 ± 1.1	98.6 ± 0.8	98.0 ± 1.2	98.0 ± 1.2
AA2BR(83)	97.6 ± 1.0	98.2 ± 0.7	99.4 ± 0.6	99.4 ± 0.6	99.4 ± 0.6
AA3R(17)	97.5 ± 2.2	82.5 ± 1.8	94.2 ± 3.2	95.0 ± 4.5	95.0 ± 4.5
CLTR1(18)	89.2 ± 2.5	84.2 ± 4.1	89.2 ± 4.3	91.7 ± 3.1	91.7 ± 3.1
LT4R1(2)	50.0 ± 25.0	50.0 ± 25.0	100.0 ± 0.0	100.0 ± 0.0	100.0 ± 0.0
LT4R2(2)	50.0 ± 25.0	50.0 ± 25.0	100.0 ± 0.0	100.0 ± 0.0	100.0 ± 0.0
MTR1A(91)	97.3 ± 1.1	96.8 ± 1.4	100.0 ± 0.0	100.0 ± 0.0	100.0 ± 0.0
MTR1B(90)	97.8 ± 0.9	97.8 ± 0.9	99.4 ± 0.5	99.4 ± 0.5	99.4 ± 0.5
MTR1L(75)	98.7 ± 0.7	99.3 ± 0.6	100.0 ± 0.0	100.0 ± 0.0	100.0 ± 0.0
PAFR(1)	50.0 ± 35.4	50.0 ± 35.4	50.0 ± 35.4	50.0 ± 35.4	50.0 ± 35.4
PE2R1(5)	100.0 ± 0.0	100.0 ± 0.0	100.0 ± 0.0	100.0 ± 0.0	100.0 ± 0.0
PE2R2(7)	100.0 ± 0.0	95.0 ± 4.5	100.0 ± 0.0	100.0 ± 0.0	100.0 ± 0.0
PE2R3(5)	100.0 ± 0.0	100.0 ± 0.0	100.0 ± 0.0	100.0 ± 0.0	100.0 ± 0.0
PE2R4(5)	100.0 ± 0.0	100.0 ± 0.0	100.0 ± 0.0	100.0 ± 0.0	100.0 ± 0.0
R3R2(1)	50.0 ± 0.0	100.0 ± 0.0	100.0 ± 0.0	100.0 ± 0.0	100.0 ± 0.0
TA2R(63)	100.0 ± 0.0	99.2 ± 0.7	99.2 ± 0.7	100.0 ± 0.0	100.0 ± 0.0
		Metabotropic glutamate family			
GABR1(1)	50.0 ± 0.0	100.0 ± 0.0	100.0 ± 0.0	50.0 ± 35.4	100.0 ± 0.0
GABR2(1)	50.0 ± 0.0	100.0 ± 0.0	100.0 ± 0.0	0.0 ± 0.0	50.0 ± 35.4
MGR1(34)	98.3 ± 1.5	91.4 ± 4.7	100.0 ± 0.0	100.0 ± 0.0	100.0 ± 0.0
MGR2(6)	95.0 ± 4.5	100.0 ± 0.0	100.0 ± 0.0	100.0 ± 0.0	100.0 ± 0.0
MGR3(5)	100.0 ± 0.0	90.0 ± 8.9	100.0 ± 0.0	100.0 ± 0.0	100.0 ± 0.0
MGR5(5)	90.0 ± 8.9	100.0 ± 0.0	100.0 ± 0.0	100.0 ± 0.0	100.0 ± 0.0
MGR6(5)	100.0 ± 0.0	90.0 ± 8.9	90.0 ± 8.9	100.0 ± 0.0	90.0 ± 8.9
MGR7(6)	95.0 ± 4.5	90.0 ± 8.9	100.0 ± 0.0	100.0 ± 0.0	100.0 ± 0.0
MGR8(3)	80.0 ± 17.9	80.0 ± 17.9	100.0 ± 0.0	100.0 ± 0.0	100.0 ± 0.0
		Secretin family			
VIPR1(1)	50.0 ± 35.4	100.0 ± 0.0	100.0 ± 0.0	50.0 ± 35.4	100.0 ± 0.0
